# Acute Effects of Different Postactivation Potentiation Protocols on Traditional Rowing Performance

**DOI:** 10.3390/ijerph18010080

**Published:** 2020-12-24

**Authors:** Alfonso Penichet-Tomas, Jose M. Jimenez-Olmedo, Luis Serra Torregrosa, Basilio Pueo

**Affiliations:** Department of General and Specific Didactics, University of Alicante, 03690 Alicante, Spain; alfonso.penichet@ua.es (A.P.-T.); lst15@gcloud.ua.es (L.S.T.); basilio@ua.es (B.P.)

**Keywords:** sprint, postactivation potentiation, fixed seat rowing, performance

## Abstract

Postactivation potentiation (PAP) describes an initial muscular activation with a submaximal or maximal load intensity that produces acute improvements in muscle power and performance in subsequent explosive activities. The objective of this study was to compare the effect of different PAP protocols in rowing performance. A crossover design involving seven rowers was used, in which two different PAP protocols were applied: PAP of maximal conditioning contractions (PAP MCC) on a rowing ergometer to provide greater transferability and, thus, enhance the magnitude of PAP stimuli on subsequent rowing performance; and PAP of maximal strength contractions (PAP MSC) in half squat and bench pull exercises, similar to the main exercises in rowing strength training, to perform a 20 s “all-out” test simulating a competition start. Student’s *t*-test was used to compare means of the variables (*p* < 0.05). Effect size statistics were calculated using Cohen’s *d*. The PAP MCC protocol resulted in significant differences, with an extremely large effect size in average power output (*p* = 0.034, *d* = 0.98) in the first 3 (*p* = 0.019, *d* = 1.15) and first 5 (*p* = 0.036, *d* = 0.91) strokes. This group also reached a greater number of strokes (*p* = 0.049, *d* = 2.29) and strokes per minute (*p* = 0.046, *d* = 1.15). PAP with maximal conditioning contractions in rowing warm-up enhanced subsequent rowing sprint and is an advisable strategy to potentiate performance at the start of rowing competitions and sprint regattas.

## 1. Introduction

Postactivation potentiation (PAP) describes an initial muscular activation with a submaximal or maximal load intensity that produces acute improvements in muscle power and performance in subsequent explosive activities [1]. This procedure involves concentric contractions of submaximal dynamic force close to a high percentage of maximal repetition (1RM) before the execution of an explosive movement with similar movement patterns. However, if the load induced by the PAP is poorly planned in relation to the volume or intensity of work, it can cause fatigue. Therefore, the balance between PAP and fatigue is vital because of its role in the magnitude of improvement in mechanical power [2]. Determination of the characteristics in relation to the components of the load (volume, intensity, density, and type of exercise) are key to the subsequent performance induced by the PAP [3].

The optimal intensity of the conditioning activities for competitive athletes varies between 75% and 90% of 1RM, and recovery time should be individualized because athletes differ in strength level, training experience, and muscle fiber structure, with the optimal recovery time being 6 min [4]. Stronger recreational level athletes would experience greater benefit with shorter breaks (5–10 min) than weaker athletes, who would need more time (15–20 min) [5]. Results of this study suggest that the principle of individualization is key to determining the rest time necessary for optimal activation performance following PAP protocols. Seitz et al. [6] observed increased force production with activation time after PAP in elite athletes. Coaches must consider individual differences in the strength level(s) of athletes to adapt protocols and maximize performance effects. Esformes et al. [7] related the difference in muscle fiber activation according to the type of contraction with rest time after PAP. They concluded that it could positively influence power performance; however, in relation to the level of activation and type of contraction, there were only significant differences in isometric contractions with a long rest period (12 min), producing positive results for peak power output, peak force, maximum distance and rate of force development, and inducing a longer PAP period. On the other hand, the application of a previous PAP methodology involving maximal dynamic force with submaximal loads close to 1RM in half squat exercises can be very useful for improving performance in the ability to repeat high-intensity sprints [8]. Furthermore, PAP would be beneficial in events where the contribution of the aerobic energy system may be of high importance toward successful performance; however, research in this area is quite limited. Silva et al. [9] found that heavy strength exercise bouts improve 20 km cycling time trial performance with no alteration in pacing strategy. In rowing, PAP could improve the efficiency of the working muscles by increasing force production of muscle fibers if a specific pace is to be maintained [10].

Strength training in elite rowers represents 10–20% of the total training time. During the competitive season, rowers perform strength training with a load of between 85% and 95% of 1RM [11]. There is a strong relationship between a high manifestation of strength and greater performance [12]. Doma et al. [2] demonstrated that maximal dynamic contractions on a rowing machine increased power performance during the execution of a short sprint (10 s). In addition, the average power during a 20 s maximal effort test is an effective predictor of 2000 m Olympic rowing performance [13]. Nevertheless, traditional rowing is characterized, and differs from Olympic rowing, in technical execution because the rower is supported by a fixed seat in the coccyx area [14]. This implies that the degree of trunk amplitude both in the attack and final phases is greater than in Olympic rowing. Studies on traditional rowing have evaluated physiological factors as predictors of performance [15] as well as specific protocols for improving sports performance [16]. On the other hand, assessment and analysis strategies derived from Olympic rowing have been developed [17] to be able to adapt to the needs of traditional rowing [18], leading to comparative studies between both disciplines [19]. The scientific literature also includes studies in the area of traditional rowing on specific and conditional physiological requirements, quantifying the workload in official competition [20]. Accordingly, there are studies on the relationship between the characteristics of rowers and their sports performance [21], sports practice habits and quantification of training hours [22], as well as profile analysis of rowers using traditional rowing modalities based on the competitive level [14].

The PAP is considered to be an adequate specific warm-up by coaches in both day-to-day training or competition, and current scientific evidence shows that there is a positive effect on performance when using PAP in short sprint efforts [1]. In addition, the greatest response is manifested in experienced subjects in high-intensity training [23]. However, there is no current scientific evidence supporting intervention studies that have proposed the comparison of the effects of different PAP types on rowing sprint performance, a sport in which physical and physiological factors, such as aerobic and anaerobic power, influence as well as a high level of physical strength [24]. In addition, no study has analyzed the influence of PAP using a traditional rowing modality. Therefore, the objective of this study was to analyze and compare the acute effect of two different postactivation potentiation protocols in traditional rowing performance at the start of competition. The strength contraction with maximum loads in strength exercises of the main muscle groups in the rowing gesture are an effective PAP method for rowing performance. However, maximum effort power conditioning contractions will better prepare the rower as this PAP method increases neuromuscular performance and has a greater dynamic transfer because it involves a biomechanically similar exercise, and rowers perform more effective neural patterns enhancing a more specific gesture.

## 2. Materials and Methods

### 2.1. Experimental Design

**Figure 1:**
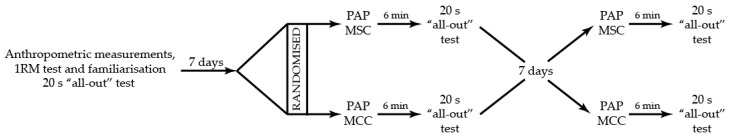
Schematic representation of the study design.

A crossover design was used to compare the effect of both PAP protocols: PAP of maximal conditioning contractions (PAP MCC) and PAP of maximal strength contractions (PAP MSC). In this repeated measurement design, each participant performed the different protocols during different time periods (i.e., the participants crossed over from one protocol to another during the research process). The reason a crossover design was considered was that it could yield a more efficient comparison of treatments than a parallel design (i.e., fewer participants might be required in the crossover design to attain the same level of statistical power or precision as a parallel design). Hence, each participant served as his own matched control and performed both protocols: PAP MCC and PAP MSC [25]. Participants were tested three times in three consecutive weeks on the same day of the week at the same time. The first session was oriented to familiarize the athletes with the techniques and methods, and the next weeks’ session addressed the 2 PAP protocols (Figure 1). The protocols were assessed in randomized order with a one-week interval in between.

### 2.2. Participants

Seven male rowers, competing at the national level, participated in the study. Inclusion criteria for the present study consisted of a minimum previous experience of 2 consecutive full years in traditional rowing, with a minimum of 10 months per year and an average of 12 h per week [17]. Rowers who did not meet the selection criteria were excluded from the study. Table 1 summarizes the anthropometric characteristics of the sample.

The Ethics Committee at the University of Alicante gave institutional approval for this study, in accordance with the Declaration of Helsinki (IRB UA-2020-07-21). The subjects were informed about the study and gave their written informed consent. They were notified of the need for their commitment to the following requirements on the day of data collection and the previous day: not to perform high-intensity physical activity in the previous 24 h; not to consume any type of stimulant at least 5 h before testing; and not to eat any solid food at least 3 h before testing.

### 2.3. Procedures

Data collection was performed for 3 weeks on the same day of the week at the same time each week. The first week was used to perform the test of maximal repetition (1RM) in half squat and bench pull [26], to perform anthropometric measurements, and to familiarize the athletes with the 20 s “all-out” test. Anthropometric measurements were performed in basal conditions following standard protocols from the International Society for the Advancement of Kinanthropometry (ISAK) [27] using the following instruments: a TanitaBC-545N balance for body mass (0.1 kg) (Tanita Co., Tokyo, Japan) [28] and an astra stadiometer with a mechanical scale to measure height (0.1 cm). Body mass index (BMI) was computed as body mass (kg) divided by height squared (m^2^) [29]. Skinfolds (sub-scapular, tricipital, bicipital, iliac crest, supra-spinal, abdominal, anterior thigh, and middle leg) were measured using a caliper calibrated to the nearest 0.2 mm (Holtain Ltd., Crymych, UK). Girth (relaxed arm, flexed arm, thigh, and calf) and breadth (humerus, stylion, and femur) measurements were performed using a flexible anthropometric steel tape to the nearest 0.1 cm (Holtain Ltd., Crymych, UK). Finally, body composition was calculated using the equations described by Withers, Craig, Bourdon, and Norton [30] for fat mass and the equation proposed by Lee et al. [31] for muscle mass.

After warm-up, the 1RM test consisted of two sets of 5 and 3 repetitions at approximately 50% and 70% 1RM, respectively. The subjects then had up to 5 attempts to obtain their 1RM [8]. A 3–5 min rest interval was adopted between trials. Each attempt consisted of 1 series of 1 repetition. If the attempt was successful, between 5% and 10% of the weight was added for the next attempt. When the subject could not perform the execution properly, until the subject was unable to lift the load, his previous attempt was his 1RM.

**Figure 2:**
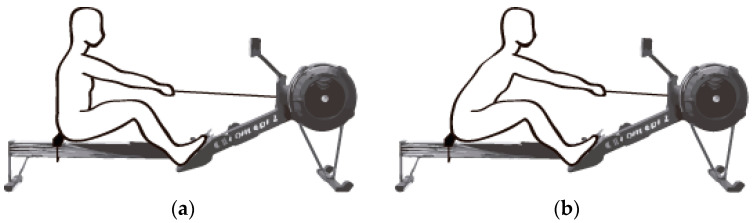
Differences in technique for (**a**) the first 3 strokes and (**b**) after the first 3 strokes.

After performing the 1RM tests, the familiarization test was performed for proper technical execution of the 20 s “all-out” test. The subjects performed three sets of 20 s at maximal with a rest interval of 6 min to simulate the intensity on test days. The test simulated a start; as such, in the first 3 strokes, the shoulders started from a vertical position of the body (Figure 2). After the first 3 strokes, the objective would be to achieve the maximal possible power in 20 s with a correct wide stroke and with a maximal ratio of strokes per minute.

During the second week, data were collected according to the crossover design in which the subjects were distributed in two groups: one performed the PAP MCC protocol and the other performed the PAP MSC protocol. In the third week, the groups rotated to perform the protocol they did not perform the week before. First, both groups performed 5 min of warm-up on the rowing machine (Concept 2 Model D, USA, Vermont) [11] with a coupling adapted for the reproduction of the traditional rowing stroke, programmed with a drag factor of 160, a given power (up to a maximal of 140 W or 2:15 / 500 m), and at a heart rate (HR) not exceeding 140 beats/min [17].

After the warm-up, each group performed a different method of PAP in each session before the 20 s “all-out” test, with a 6 min rest before and after the PAP protocol. The PAP MCC protocol consisted of performing a series of 20 s at maximal effort on the rowing machine before performing the 20 s “all-out” test. In Doma et al. [2], a 10 s maximal dynamic conditioning contraction protocol was executed on a rowing ergometer at maximal effort, followed by a 10 s “all-out” test. In the present study, the 20 s “all-out” sprint test was performed because it covered the 10 s time span and could yield additional information. In addition, a 20 s “all-out” test can be an effective predictor of the performance in a 2000 m test [13]. The rest interval between the PAP and the post-test was 6 min [32].

The athletes performed the PAP MSC protocol in the two exercises performed in the 1RM test, with a protocol of 4 series of submaximal approach (1 × 5 with the bar/2 min, 1 × 2 50% RM/4 min, and 1 × 1 85% RM) and 1 set of 3 repetitions at 90% of 1RM [33]. The rest interval prior to the 20 s “all-out” test was also 6 min [32]. The intensity of the exercise that elicits the greatest PAP effect should be individualized (60–100% 1RM) because it is dependent on the level of maximal strength [34]. The 20 s “all-out” test was performed on a rowing machine (Concept 2 Model D, Morrisville, VT, USA) [11] with a coupling adapted for traditional rowing fixing the seat. The measurement of performance variables was achieved using the Erg Data mobile device application in sync with the PM5 performance monitor of the rowing machine via Bluetooth Smart. Heart rate parameters were measured with the Polar M400 (Polar Inc., Kempele, Finland) with H7 Bluetooth^®^ Smart Band [8].

### 2.4. Statistical Analysis

Results were analyzed using Statistical Package for Social Sciences (SPSS v.26 for Windows, SPSS Inc., Chicago, IL, USA), and values of each variable are expressed as mean and standard deviation. With the aim to determine whether the quantitative variables maintained the criterion of normality, a Shapiro–Wilk statistical test was performed. Fulfilling the criteria of normality, Student’s *t*-test was used to compare the means of the variables (*p* < 0.05) pairwise between PAP MCC and PAP MSC. Cohen’s *d* was used as a measure of the effect size of differences between protocols and interpreted according to Cohen’s thresholds: small (*d* < 0.3), medium (*d* = 0.3–0.4), large (*d* = 0.5–0.6), very large (*d* = 0.7–0.9), and extremely large (*d* > 0.9) [35].

## 3. Results

When comparing performance parameters between PAP MCC and PAP MSC, the power outputs of the PAP MCC group were highest in the 20 s “all out” test. Significant differences with an extremely large effect size were noted in average power output (*p* = 0.034, *d* = 0.98), power output in the first 3 strokes (*p* = 0.019, *d* = 1.15), and in the first 5 strokes (*p* = 0.036, *d* = 0.91) (Table 2). Higher values and very large effect size were reached in maximal power output (*p* = 0.080, *d* = 0.74) and first stroke power output (*p* = 0.085, *d* = 0.84), although no significant differences were found.

The total number of strokes (*p* = 0.049, *d* = 2.29) and the rate of strokes per minute (*p* = 0.046, *d* = 1.15) were significantly greater in the PAP MCC group than in the PAP MSC group, with an extremely large effect size.

Both average and maximum heart rates were also higher in the PAP MCC group than in the PAP MSC group, but no significant differences were found in heart rate difference. The effect size for average heart rate was extremely large (*p* = 0.050, *d* = 1.40), whereas there was a very large effect size for maximum heart rate (*p* = 0.225, *d* = 0.78).

## 4. Discussion

The objective of this study was to analyze and compare the acute effects of two different PAP protocols in traditional rowing performance using a crossover design such that each participant performed different protocols during different time periods. In the current study, we sought to determine the PAP protocol that would be most beneficial for rowing performance. The group with the highest values of power output reached in the 20-s “all-out” test was the group that performed the PAP of maximal conditioning contractions (PAP MCC) in rowing ergometer compared with the group that performed the PAP of maximal strength contractions (PAP MSC) in half squat and bench pull. Doma et al. [2] found higher power values in Wmean, Wmax, and W_1_stroke with the rowers who performed dynamic conditioning contractions before the test. In the same way, isometric conditioning contractions on a rowing ergometer to the rowing warm-up appears to increase short-term rowing ergometer performance, especially at the start [10]. The effect of the postactivation potentiation of conditioning contractions, with similar movement patterns of rowing gesture, enhances power output. However, the type of conditioning contractions may have different effects on PAP and fatigue. Isometric conditioning contractions may induce central fatigue, while dynamic conditioning contractions may induce the opposite response [36].

Prior heavy load exercise will induce some activation adaptations to the nervous system that will help increase performance in the subsequent test(s) as long as the recovery time is adequate [8]. However, the PAP MSC group reached the lower values in all measured power variables. This may indicate that this type of PAP may not benefit rowing performance. The greatest adaptations will occur in experienced athletes performing high-intensity training [23]; therefore, they would need a longer recovery time after PAP [5]. In a study by Esformes et al. [7], greater manifestation of the PAP MSC was also related to a shorter recovery time after the PAP. In this way, if the recovery time after the PAP is not sufficient, it will cause fatigue conditioning in subsequent efforts. Both the degree of PAP MSC and the recovery time after a PAP will be linked to performance optimization. Therefore, the degree of manifestation of the maximum dynamic strength must be taken into account individually to quantify the recovery time after the PAP correctly [6].

The increase in power achieved with the PAP MCC protocol may have explained the faster average stroke rate and the total number of the strokes, both in the present study performed in a 20 s “all-out” test and in other studies evaluated over 1000 m rowing ergometer time trial [10] and over a 10 s “all-out” test [2].

The mean and maximum heart rate values were higher in the PAP MCC group. When a very explosive test is carried out, HR increases very quickly when it comes from a 6 min recovery where it practically returns to a resting HR range. The PAP MCC group previously performed the PAP protocol, and the heart had already reached a working HR range. The same did not occur with the PAP MSC group because during the PAP protocol, they did not reach such high HR values. Therefore, when the test was performed, the HR did not increase so fast because HR is a component of late physiological response or due to a possible greater activation of the sympathetic system after conditioning contractions versus strength contractions [37].

Regarding limitations, findings from the present study should be interpreted with caution due its to the small sample size, although a crossover design was used. Furthermore, the results should be interpreted considering that the study was performed on a rowing start and not on competitive distance, an event in which contribution(s) of the aerobic energy system may be highly important to successful performance. Future research aimed at confirming our results in a larger population and using a 2000 m rowing performance test, and not only in performance at the rowing start, would be interesting. Additionally, training using a 1RM load is inconsistent with safety rules. A better solution to describe the 1RM movement technique is to work at a specific rate of movement, describing the tempo of phases of movement (e.g., eccentric, transition, and concentric) [38].

## 5. Conclusions

The results suggested that postactivation potentiation with maximal conditioning contractions in rowing warm-up enhanced subsequent rowing sprint performance, simulating the start of a race in traditional rowing. It can be an interesting strategy to potentiate rowing performance if prescribed by coaches before activities that involve explosive starts; as such, rowing coaches should implement this form of training.

Coaches can prescribe maximal conditioning contractions in warm-ups before starting specific rowing training as well as in competitions. However, more research is needed to determine whether this PAP protocol and other methodologies can also improve 2 km rowing performance.

## Figures and Tables

**Table 1:** Anthropometric characteristics.

	Mean ± SD	95% CI
Body height (cm)	181.6 ± 5.8	178.1–185.0
Body mass (kg)	76.1 ± 4.4	73.5–78.7
BMI (kg/m^2^)	23.1 ± 1.4	22.3–23.9
Fat mass (%)	10.5 ± 2.0	9.3–11.5
Muscle mass (%)	46.5 ± 2.0	45.3–47.7

BMI: body mass index; SD: standard deviation; CI: confidence interval.

**Table 2:** Results of the 20 s sprint test “all-out”.

	PAP MCC	PAP MSC	*p*	95% CI	Effect Size	
	Mean ± SD	Mean ± SD			*d*	Size
Wmean (W)	554.3 ± 30.5	514.5 ± 48.9	0.034 *	4.6–96.7	0.98	Extremely large
Wmax (W)	621.4 ± 54.9	582.8 ± 48.7	0.080	−6.9–106.0	0.74	Very large
W_1_stroke (W)	251.4 ± 63.0	211.0 ± 25.2	0.085	−11.0–133.0	0.84	Very large
W_3_strokes (W)	470.2 ± 64.3	392.5 ± 70.2	0.019 *	18.5–171.0	1.15	Extremely large
W_5_strokes (W)	600.4 ± 43.7	562.0 ± 40.9	0.036 *	4.1–102.8	0.91	Extremely large
Strokes (n)	17.2 ± 1.6	15.2 ± 1.5	0.049 *	0.0–3.7	1.29	Extremely large
Ratio (stroke/min)	49.9 ± 4.4	44.8 ± 4.5	0.046 *	0.1–11.5	1.15	Extremely large
HRmean (bpm)	118.5 ± 9.1	101.2 ± 14.9	0.050	0.0–34.7	1.40	Extremely large
HRmax (bpm)	129.8 ± 11.9	115.3 ± 23.4	0.225	−10.9–38.9	0.78	Very large

PAP: postactivation potentiation; MCC: maximal conditioning contractions; MSC: maximal strength contractions; SD: standard deviation; CI: confidence interval; W: watt; max: maximum; N: number; min: minute; HR: heart rate; *: statistically significant (*p* < 0.05).
